# Postoperative mortality and complications in patients with and without preoperative SARS-CoV2 infection: A service evaluation of 24 million linked records using OpenSAFELY

**DOI:** 10.1111/anae.16001

**Published:** 2023-03-23

**Authors:** C. D. McInerney, A. Kotzé, S. Bacon, J. E. Cutting, L. Fisher, J. Kua, D. McGuckin, A. Mehrkar, B. Goldacre, The OpenSAFELY Collaborative, O.A. Johnson, S.R. Moonesinghe

**Affiliations:** 1 Academic Unit of Primary Medical Care, University of Sheffield, Sheffield, England; 2 School of Computing, University of Leeds, Leeds, England; 3 National Institute for Health Research Yorkshire and Humber Patient Safety Translational Research Centre; 4 Leeds Teaching Hospitals NHS Trust, Leeds, England; 5 School of Medicine, University of Leeds, Leeds, England; 6 Bennett Institute for Applied Data Science, Nuffield Department of Primary Care Health Sciences, University of Oxford, OX2 6GG, UK; 7 Gloucestershire Royal Hospitals NHS Foundation Trust, Gloucestershire, England; 8 Department of Targeted Intervention, Centre for Perioperative Medicine, Division of Surgery and Interventional Science, University College London, London, UK; 9 School of Computing, University of Leeds, Leeds, England; 10 Research Department of Targeted Intervention, Centre for Perioperative Medicine, Division of Surgery and Interventional Science, University College London, London, UK

## Abstract

Surgical decision making after SARS-CoV-2 infection is influenced by the presence of comorbidity, infection severity, and whether the surgical problem is time-sensitive. Contemporary surgical policy to delay surgery is informed by highly heterogenous country-specific guidance. We evaluated surgical provision in England during the COVID-19 pandemic to assess real-world practice and whether deferral remains necessary. Using the OpenSAFELY platform, we adapted the COVIDSurg protocol for a service evaluation of surgical procedures that took place within the English National Health Service (NHS) from 17 March 2018 to 17 March 2022. We assessed whether hospitals adhered to guidance not to operate on patients with seven weeks of an indication of SARS-CoV-2 infection. Additional outcomes were postoperative all-cause mortality (30-day, 6-month), and complications (pulmonary, cardiac, cerebrovascular). The exposure was the interval between the most-recent indication of SARS-CoV-2 infection and subsequent surgery.

In any 6-month window, less than 3% of surgical procedures were conducted within seven weeks of an indication of SARS-CoV-2 infection. Mortality for surgery conducted within two weeks of a positive test in the era since widespread SARS-CoV-2 vaccine availability was 1·1%, declining to 0·3% by four weeks. Compared to the COVIDSurg study cohort, outcomes for patient in the English NHS cohort were better during the COVIDSurg data collection period and the pandemic era before vaccines became available. Clinicians within the English NHS followed national guidance by operating on very few patients within seven weeks of a positive indication of SARS-CoV-2 infection. In England, surgical patients’ overall risk following an indication of SARS-CoV-2 infection is lower than previously thought.

Surgical decision making after SARS-CoV-2 infection is influenced by the presence of comorbidity, infection severity, and whether the surgical problem is time-sensitive [1]. The COVIDSurg collaborative conducted the largest prospective study on surgical outcomes after SARS-CoV-2 infection to date, showing increased postoperative mortality and pulmonary complications up to seven weeks following a positive test in patients who have recovered fully [2]. This study was conducted before vaccines [3] or evidence-based drug therapy for severe COVID [4] became available. However, United Kingdom (UK) [5] and German [6] guidance still recommends deferring deferrable elective surgery for seven weeks after SARS-CoV-2 infection. In contrast, current guidance in the United States of America (USA) recommends seven weeks’ postponement in unvaccinated individuals only [7]. Guidance from Australia and New Zealand recommend stratification by surgical magnitude, with postponement ranging from four weeks for minor, to 12 weeks in major surgery [8].

Contemporary surgical policy-making is, therefore, constrained by very heterogenous global guidance. A paucity of studies in highly-vaccinated populations has been identified as a limiting factor for evidence-based policy making in the USA [7], Germany [6] and Australia and New Zealand [8]. It is also unclear as to what extent the above guidance is followed, and how possible variance in enactment is associated with outcomes. Scheduling constraints might also be a factor, limiting the capacity of health services to clear the post-pandemic backlog of cancer and other elective surgery [7,9].

Postponement of potentially curative cancer surgery can worsen overall survival. A meta-analysis conducted before 2020 found that a 12-week delay to surgery was associated with decreased overall survival in early-stage breast, lung, and colon cancer [10]. In patients with non-cancer pathology such as osteoarthritis, further postponement of surgical intervention on the background of already-long waiting lists has been identified as exposing patients to continuing suffering, potentially poorer long-term outcomes, and increased long-term opioid use [11].

In this context, we identified a need to evaluate the provision of timely and safe surgery during the COVID-19 pandemic both before and after vaccines became available. We conducted a retrospective observational study of 24 million linked primary and secondary care records across England. Our aims were to:

- Establish to what extent English hospitals scheduled surgery within 7 weeks from a SARS-CoV-2 diagnosis; *and*
- Describe post-operative outcomes, stratified by:
  - Time between surgery and a SARS-CoV-2 infection, or no infection
  - Surgery before and after widespread vaccine availability.

## Methods

We adapted the COVIDSurg protocol [2] to account for the retrospective nature of our service evaluation. The exposure was the duration between an indication of SARS-CoV-2 infection and the patient’s date of surgery. An unabridged description of our methods is given in Appendix 1.

The data source was OpenSAFELY, a secure and transparent platform linking data from two major National Health Service (NHS) primary care record providers with relevant databases from secondary care, and with the UK Office of National Statistics. Linkage and analysis are conducted within the records providers’ data centres (OpenSAFELY-EMIS and OpenSAFELY-TPP), meaning that researchers never see individual-level data. All data were linked, stored and analysed securely within the OpenSAFELY platform [12]. Data include pseudonymised data such as coded diagnoses, medications and physiological parameters. No free text data are included. Only OpenSAFELY-TPP contains data on surgical events. OpenSAFELY-TPP comprises around 24 million patient records, from over 2,600 GP practices and a third of mental health Trusts in England, and is representative of the English population [13,14]. All code is shared openly for review and re-use under MIT open license (https://github.com/opensafely/surg-covid-safely). Our study population was patients who underwent surgery between 17 March 2018 and 17 March 2022. The start and end dates were chosen as being two years before and after the date that NHS England announced the temporary postponement of all elective surgery as part of the pandemic response [15].

This study was a service evaluation with sponsorship from NHS England and additional institutional ethical approval by the University of Leeds Faculty for Engineering and Physical Sciences Ethics Committee (reference MEEC 21-005). NHS England is the data controller for OpenSAFELY-EMIS and OpenSAFELY-TPP. EMIS and TPP are the data processors. All study authors using OpenSAFELY had the approval of NHS England. This implementation of OpenSAFELY is hosted within the TPP environment, which is accredited to the ISO 27001 information security standard and is NHS IG Toolkit compliant [16].

Patient data were pseudonymised for analysis and linkage using industry standard cryptographic hashing techniques; all pseudonymised datasets transmitted for linkage onto OpenSAFELY are encrypted; access to the platform is via a virtual private network connection, restricted to a small group of researchers; the researchers hold contracts with NHS England and only access the platform to initiate database queries and statistical models; all database activity is logged; only aggregate statistical outputs leave the platform environment following best practice for anonymisation of results such as statistical disclosure control for low cell counts.

The OpenSAFELY research platform adheres to the obligations of the UK General Data Protection Regulation and the Data Protection Act 2018. In 2020, the Secretary of State for Health and Social Care used powers under the UK Health Service (Control of Patient Information) Regulations 2002 (COPI) to require organisations to process confidential patient information for the purposes of protecting public health, providing healthcare services to the public, and monitoring and managing the COVID-19 outbreak and incidents of exposure; this sets aside the requirement for patient consent [17]. This was extended in November 2022 for the NHS England OpenSAFELY COVID-19 research platform [18]. In some cases of data sharing, the common law duty of confidence is met using, for example, patient consent or support from the Health Research Authority Confidentiality Advisory Group. Taken together, these provide the legal bases to link patient datasets on the OpenSAFELY platform. GP practices, from which the primary care data are obtained, are required to share relevant health information to support the public health response to the pandemic, and have been informed of the OpenSAFELY analytics platform. The study was supported by Ramani Moonesinghe (National Clinical Director for Critical and Perioperative Care, NHS England and NHS Improvement) as senior sponsor.

Our outcomes were those used in the COVIDSurg study [2]: all-cause mortality at 30 days and six months postoperatively, as well as 30-day post-operative pulmonary, cardiac, and cerebrovascular complications. The exposure was the interval between the most-recent indication of SARS-CoV-2 infection and subsequent surgery. While COVIDSurg calculated intervals in weeks, we calculated intervals in days and modelled categorically, namely “no pre-operative indication of SARS-CoV-2 infection”, “≤14 days”, “15-28 days”, “29-42 days”, and “≥43 days”. Preoperative SARS-CoV-2 testing was mandatory in England between July 2020 [19] and April 2022 [17]. Preoperative PCR tests are conducted via the UK’s Pillar 1 (clinical need) route and no selection bias would be expected since all Pillar 1 test results are available in OpenSAFELY.

We stratified across the same concepts as the COVIDSurg study [2], except for the Revised Cardiac Risk Index where we stratified on the presence of cardiac or cerebrovascular disease. We did not construct multivariable regression models, to mitigate collider bias which has been found to be a risk in CoVID-19-related research [20].

**Figure 1:**
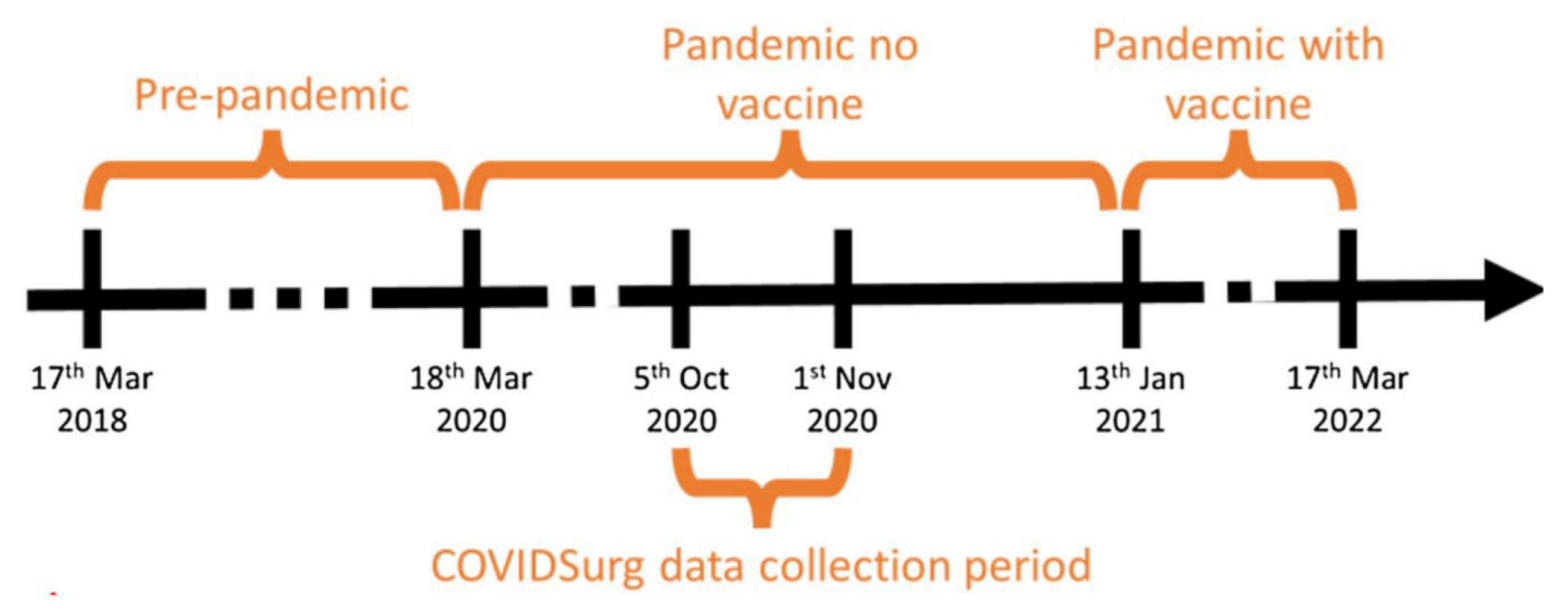
Timeline of key study dates (vertical black lines) that define study era (orange periods).

We did not query individuals’ vaccination status at the time of surgery. Given rapid vaccination uptake and high levels of coverage in England [21], we assumed that the group who are both unvaccinated and required surgery at any time point are highly likely to be atypical in unknown ways. We also wanted to diverge from previous studies that focused on infection at the individual level in an at-risk population by providing aggregate summaries of a general population, on which general public-health policy is better based. We defined three eras for stratification (figure 1):

- *Pre-pandemic*: 17 March 2018 to 17 March 2020.
- *Pandemic-no-vaccine:* 18 March 2020 to 12 January 2021. We chose 12 January 2021 as the end of the period when vaccination was unavailable, because the first vaccines were administered on 5 December 2020. After this date, we allowed 3 weeks for completing the vaccination schedule as was recommended at the time, followed by 2 weeks for effect [22].
  ∘ Within the *pandemic no-vaccine* era, we defined a four-week *COVIDSurg data collection period*: 5 October 2020 to 1^st^ November 2020, coinciding with the data collection period for the COVIDSurg study [2].
- *Pandemic-with-vaccine:* 13 January 2021 to 17 March 2022.

Our unit of analysis was the surgical procedure; patients undergoing repeat surgery during the study period were considered more than once. We conducted a complete-case analysis, recognising that excluding patients with missing data may introduce collider and other biases [20]. We calculated counts and percentages of patients in strata of our covariates to provide clinical context for the cohorts. In accordance with guidance from OpenSAFELY, all counts ≤ 7 were redacted before all remaining counts were rounded to the nearest multiple of ten. All proportions were calculated using these rounded counts. Counts used to calculate totals were summed before redaction and rounding, so the redacted-and-rounded sum of counts from intervals does not always match the redacted-and-rounded totals.

Data management was performed using open source Python (v3.8.2) and R (v4.0.2), with analysis carried out using R. Code for data management and analysis, as well as codelists, are archived online at https://github.com/opensafely/surg-covid-safely and www.opencodelists.org (Appendix 1). The OpenSAFELY platform design requires that all analyses are prespecified and all revisions and database activity are publicly available.

## Results

**Figure 2:**
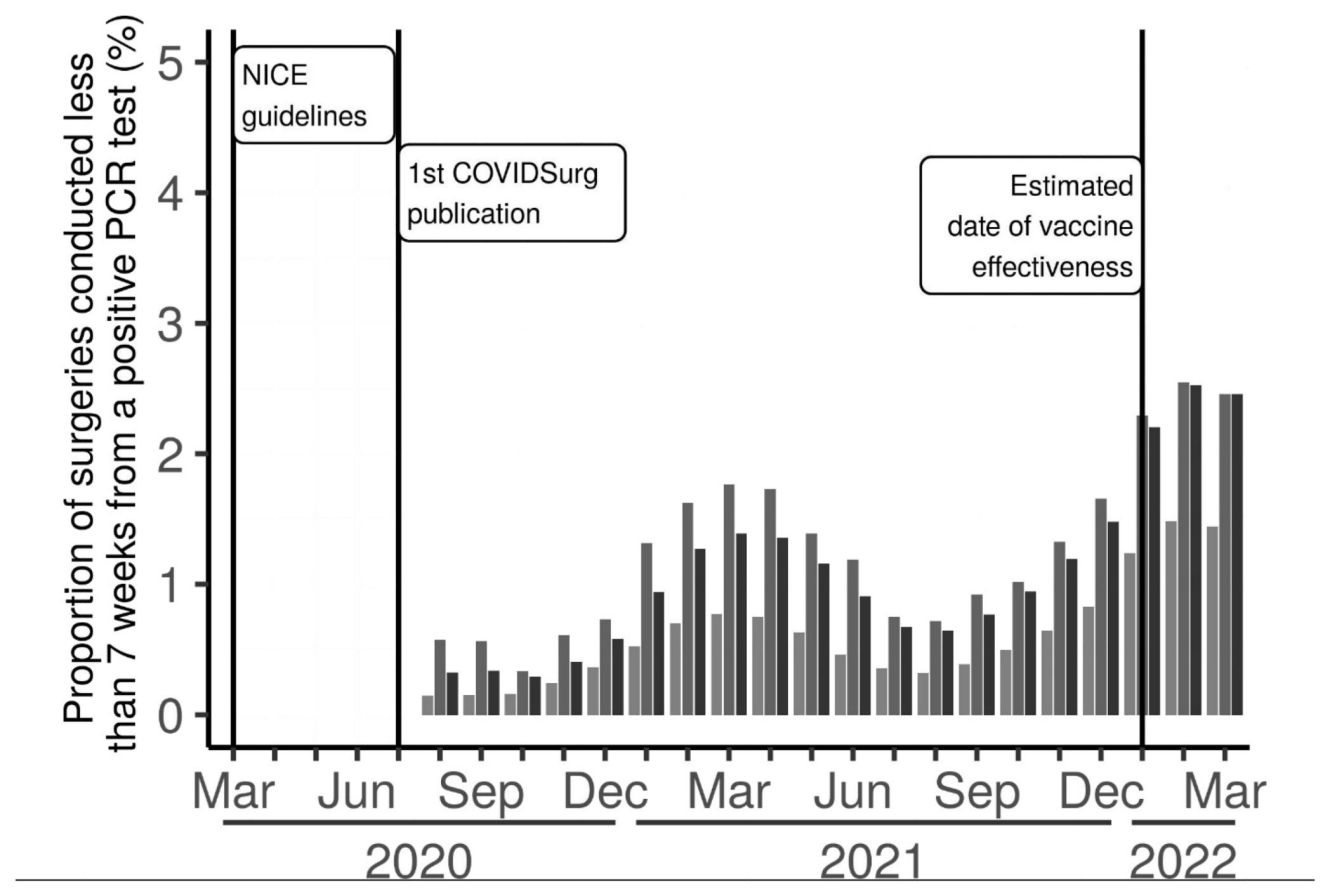
Percentage of surgeries conducted <7 weeks (<43 days) from a positive PCR assay (see caveats in section Exposure subsection of the Methods in the main text). Thin, full-length, vertical black lines indicate events of note in the timeline. The cohorts are patients who underwent surgery during elective admission (light grey) or emergency admission (dark grey), and those without a definitive admission in the record (light black).

Our analysis code was run on 15 January 2023, yielding a cohort of 3,658,140 patients undergoing surgical procedures. Of these, 1,242,180 were conducted during the pandemic-with-vaccine era on patients with a mean age of 55·1 and a standard deviation of 22·4 years. Results for other eras are given in Appendix 2. In any 6-month window, less than 3% of surgeries were conducted within the 7-week threshold after a positive PCR assay suggested by the COVIDSurg study (Figure 2). Component counts of patients in shorter intervals were so low as to breach OpenSAFELY disclosive rules. Across all time periods, a higher proportion of emergency surgery was conducted within 7 weeks of a positive SARS-CoV-2 test than elective surgery, although always less than 3% of emergency surgical caseload.

**Figure 3:**
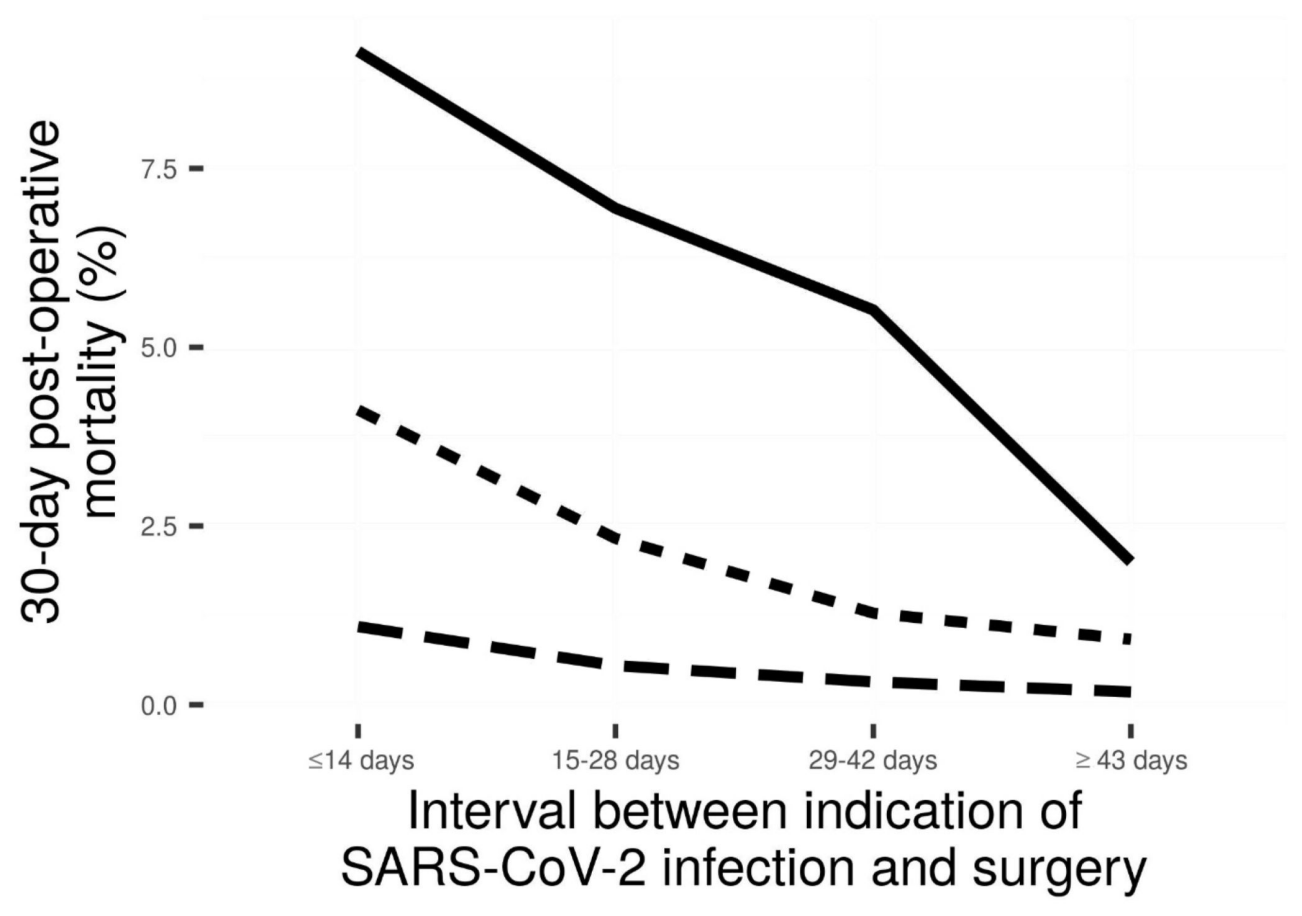
Thirty-day post-operative mortality in the COVIDSurg study (solid line), the OpenSAFELY pandemic-no-vaccine era (short-dashed line), and the OpenSAFELY pandemic-with-vaccine era (long-dashed line). OS = OpenSAFELY dataset.

Table 1 presents clinical demographics and Table 2 presents patient outcomes of the patient cohort during the pandemic-with-vaccine era. In addition to the reported outcomes, we planned to stratify by age as well as test-to-surgery interval. The returned counts were so low for younger patients that they required redaction. Overall, 30-day post-operative mortality was <0·2% and 30-day post-operative complications were <1·0%. Mortality for surgery conducted within two weeks of a positive test in the pandemic-with-vaccine era was 1·1% (compared to 9·1% in COVIDSurg), declining to 0·3% by four weeks (6·9% in COVIDSurg). Compared with the COVIDSurg study cohort, outcomes in this OpenSAFELY cohort were better during the COVIDSurg data collection period and the pandemic-no-vaccine era (Figure 3).

## Discussion

We describe the service provided by the English NHS during the COVID-19 pandemic with a focus on the extent to which guidance was followed. We described the percentage of surgical procedures conducted less than seven weeks from a positive PCR assay, and post-operative outcomes before and during the COVID-19 pandemic. Our service evaluation suggests that hospitals in England operated on very few patients within seven weeks of a positive SARS-CoV-2 test (<3% of procedures between March 2020 and March 2022). We conclude that patient outcomes were better for patients receiving care in hospitals in England than the COVIDSurg global average. The group of patients operated on within seven weeks of a positive test were so few that making risk models and further stratification would be unreliable. While the most recent UK guidelines suggest a risk-based approach to timing of surgery after SARS-CoV2 infection, clinical experience suggests that, for all but the most urgent elective or emergency surgery, clinicians continue to postpone operations if they are scheduled within 7 weeks of an indication of SARS-CoV-2 infection. If, as our data suggest, the risk associated with surgery after indication of SARS-CoV-2 infection is much lower than previously thought, delaying surgery might cause more harm than good, particularly in patients who have already waited longer than desirable for surgery.

Our findings differ from those of the COVIDSurg study [2] and subsequent UK consensus guidance [5]. Outcomes among our sample of operated patients in England were substantially better than in the COVIDSurg global surgical sample. However, even before the COVIDSurg study, very few procedures in our cohort were conducted within seven weeks of a positive test, making it likely that the cohort who were operated upon were highly selected for surgical urgency, low risk, or both.

Our findings are somewhat congruent with recent US studies on surgery in partially-vaccinated cohorts. A retrospective analysis of 228,643 patients (age: mean = 56.3; SD = 16·7) found that, compared to a pre-pandemic group, there was a greater risk of postoperative pulmonary complications in patients not completing a primary vaccination schedule before surgery and having surgery within four weeks of a positive test [23]. This was not observed in patients who underwent surgery more than 4 weeks since a positive test, nor in vaccinated patients regardless of duration since a positive test. A propensity-matched case-control study of partially- and un-vaccinated elderly American veterans (age: median = 72 and 71, respectively) undergoing surgery, observed that patients who were partially vaccinated experienced fewer COVID-19 infections, pulmonary complications, and thromboembolic events [24]. Neither of these studies provide justification for their statistical adjustments [20]. In contrast, we did not assume that completing a primary schedule confers a “vaccinated” status to stratify upon. It is recognised that repeated vaccination is required to maintain the varied protection against severe COVID-19 via a vaccine route [25,26] and any apparent relationship between individual vaccination status and outcome is likely to be confounded by the growing proportion of patients receiving both vaccinations and sustaining repeated infection, changes in the predominant virus variants, and improved treatment for severe COVID-19 [5].

In a diversion from the COVIDSurg Collaborative studies, we did not undertake regression analysis, to avoid the possibility of the so-called “Table 2 Fallacy” where biases are introduced by the analysis [25]. This has previously been an issue necessitating reversal of French national policy based on OpenSAFELY data [26]. We provided our stratification table similar to COVIDSurg Collaborative only as a benchmark for comparison under similar biases: that is, we conducted an analysis with similar biases, but with different data. We also did not attempt to delineate a subgroup who remained symptomatic after seven weeks, since the coding of “long COVID” in primary care has been found to be highly subjective, lower in OpenSAFELY-TPP than OpenSAFELY-EMIS, and much lower than in symptom prevalence surveys [27]. Primary research would be needed to describe the relationship between ongoing COVID-19 symptoms and surgical outcomes.

Our study is the largest cohort study on the relationship between an indication of SARS-CoV-2 infection and surgery to date, and includes both the eras before and after vaccination was available. It is modelled on previous work, enabling comparison. Our analyses are highly transparent and reproducible; where we have deviated from our prespecified analyses is reported in the manuscript and discoverable via our publicly-available code. The work also has important limitations. We only used records with no missing data, even though this can induce collider bias via cohort selection (20). The OpenSAFELY platform was instrumental in facilitating the analyses we conducted. Unfortunately, the approach of bringing the analysis to the data rather than the data to the analyst means that it is not possible to undertake thorough evaluations of data quality in a domain with significant data-quality challenges [28].

Our results should be interpreted with some caution. The study was a service evaluation rather than generalisable research and, as such, should not be used to infer similarity to cohorts other than surgical patients within the English NHS. We recommend that other countries evaluate their surgical services to assess whether country-specific guidelines were followed, and whether interventions are still appropriate. Our findings should not be used to guide decision-making for higher-risk groups e.g., those who remain symptomatic beyond the acute phase of COVID-19, or those individuals who are immunosuppressed, because our statistics are aggregate summaries of the patient population. Pragmatic, individualised, shared decision-making remains necessary. Furthermore, although our code lists mapped well to pre-pandemic ecological analysis of surgical activity [see supplementary material], this does not represent a comprehensive view of all surgery in England. Our results are also presented unadjusted by design since statistical adjustment in the absence of a causal model may introduce bias rather than ameliorate it [20, 29].

In conclusion, this is the first large-scale analysis of surgical outcomes throughout the COVID-19 pandemic timeline. It suggests that, in the English NHS, surgical patients’ overall risk following an indication of SARS-CoV-2 infection may be lower than previously thought. Clinicians followed national guidance by operating on very few patients within seven weeks of a positive indication of SARS-CoV-2 infection from PCR assays. Across all eras of the pandemic to date, surgical outcomes were substantially better than previously thought, even within seven weeks of a positive test. Given that delaying surgery is likely to worsen patient outcomes in the longer term, we recommend that UK guidelines should reduce the seven-week threshold for low-risk patients who have fully recovered after a positive SARS-CoV-2 test. A simple change in emphasis could suffice e.g., suggest that surgery is delayed for no more than 2 weeks after indication of a SARS-CoV-2 infection unless there are specific circumstances that places an individual at higher risk of poor outcomes. This would bring clinical guidance on surgical timing after an indication of SARS-CoV-2 infection into line with common practice regarding other acute respiratory infections. As our study is observational, we also recommend ongoing evaluation of the effect of any policy change that may result. Our analysis scripts would be deployable to repeat in a suitable environment, creating a near-real-time monitoring system of the effect of policy change. We also recommend that other countries evaluate their surgical services to assess whether country-specific guidelines were followed, and whether interventions are still appropriate. Crucially, any change in practice needs to be in the context of a real-time evaluation as our multi-faceted understanding of the physiology and epidemiology of SARS-CoV-2 improves.

## Supplementary Material

Supplementary Material

## Figures and Tables

**Table 1:** Demographic characteristics for patients who underwent surgery stratified by duration from indication of SARS-CoV-2 infection to surgery date. Period of interest is from 12^th^ January 2021 until 31^st^ March 2022 (i.e. the *pandemic-with-vaccines* era). Values are counts (n) and percentages (%). In accordance with guidance from OpenSAFELY, all counts ≤7 were redacted before all remaining counts were rounded to the nearest multiple of ten. All proportions were calculated using these rounded counts.

		No indication of infection		Interval between indication of infection and surgery							
				≤14 days		15-28 days		29-42 days		≥43 days	
		(n = 1,121,490)		(n = 3,660)		(n = 5,480)		(n = 6,250)		(n = 105,300)	
		n (%)		n (%)		n (%)		n (%)		n (%)	
Sex											
	Female	628,340 (56·0%)		2,230 (63·4%)		3,260 (59·5%)		3,740 (59·8%)		63,740 (60·5%)	
	Male	493,140 (43·9%)		1,340 (36·6%)		2,220 (40·5%)		2,510 (40·2%)		41,570 (39·5%)	
Chronic cardiac disease											
	Yes	145,440 (13·0%)		310 (8·5%)		470 (8·6%)		520 (8·3%)		9,720 (9·2%)	
	No	976,040 (87·0%)		3,350 (91·5%)		5,010 (91·4%)		5,730 (97·7%)		95,590 (90·8%)	
Diabetes											
	Yes	203,410 (18·1%)		560 (15·3%)		780 (14·2%)		850 (13·6%)		16,460 (15·6%)	
	No	918,070 (81·9%)		3,100 (84·7%)		4,700 (85·8%)		5,400 (86·4%)		88,840 (84·4%)	
Chronic respiratory disease											
	Yes	77,760 (6·9%)		150 (4·1%)		260 (4·7%)		320 (5·1%)		5,270 (5·0%)	
	No	1,043,720 (93·1%)		3,510 (95·9%)		5,220 (95·3%)		5,930 (94·9)		100,030 (95·0%)	
Cerebrovascular disease											
	Yes	49,300 (4·4%)		100 (2·7%)		140 (2·6%)		170 (2·7%)		3,110 (3·0%)	
	No	1,072,190 (95·6%)		3,560 (97·3%)		5,340 (97·4%)		6,080 (97·3%)		102,190 (97%)	
Admission method											
	Elective	648,120 (57·8%)		1,310 (35·8%)		2,420 (44·2%)		2,910 (46·6%)		55,300 (52·5%)	
	Emergency	18,180 (1·6%)		130 (3·6%)		110 (2·0%)		120 (11·9%)		1,940 (1·8%)	
	Unknown	455,220 (40·6%)		2,220 (60·7%)		2,960 (54·0%)		3,220 (51·5%)		48,060 (45·6%)	

**Table 2:** Outcomes for patients who underwent surgery stratified by duration from indication of SARS-CoV-2 infection to surgery date. Period of interest is from 12^th^ January 2021 until 31^st^ March 2022 (i.e. the *pandemic-with-vaccines* era). Values are counts (n) and percentages (%). In accordance with guidance from OpenSAFELY, all counts ≤7 were redacted before all remaining counts were rounded to the nearest multiple of ten. All proportions were calculated using these rounded counts.

		No indication of infection		Interval between indication of infection and surgery							
				≤14 days		15-28 days		29-42 days		≥43 days	
		(n = 1,121,490)		(n = 3,660)		(n = 5,480)		(n = 6,250)		(n = 105,300)	
		n (%)		n (%)		n (%)		n (%)		n (%)	
30-day post-operative mortality											
	Alive within 30 days	1,119,280 (99·8%)		3,610 (98·6%)		5,450 (99·5%)		6,230 (99·7%)		105,120 (99·8%)	
	Dead within 30 days	2,200 (0·2%)		40 (1·1%)		30 (0·5%)		20 (0·3%)		190 (0·2%)	
6-month post-operative mortality											
	Alive within 6 months	1,106,580 (98·7%)		3,550 (97·0%)		5,370 (98·0%)		6,160 (98·6%)		104,230 (99·0%)	
	Dead within 6 months	14,910 (1·3%)		110 (3·0%)		110 (2·0%)		90 (1·4%)		1,070 (1·0%)	
30-day post-operative pulmonary complications											
	No complications	1,119,680 (99·8%)		3,600 (98·4%)		5,440 (99·3%)		6,230 (99·7%)		150,130 (99·8%)	
	Complications	1,800 (0·2%)		60 (1·6%)		40 (0·7%)		20 (0·3%)		180 (0·2%)	
30-day post-operative cardiac complications											
	No complications	1,111,290 (99·1%)		3,600 (98·4%)		5,410 (98·7%)		6,180 (98·9%)		104,560 (99·3%)	
	Complications	10,190 (0·9%)		60 (1·6%)		70 (1·3%)		70 (1·1%)		740 (0·7%)	
30-day post-operative cerebrovascular complication											
	No complications	1,1120,280 (99·9%)		3,650 (99·7%)		5,470 (99·8%)		6,240 (99·8%)		105,230 (99·9%)	
	Complications	1,210 (0·1%)		Redacted		10 (0·2%)		10 (0·2%)		80 (0·1%)	

**Table 3:** Thirty-day post-operative mortality across eras, across all intervals defined by the interval between an indication of SARS-CoV-2 infection patients’ surgery date. Values are counts of deaths (n), column totals (N), and percentages (%). In accordance with guidance from OpenSAFELY, all counts ≤7 were redacted before all remaining counts were rounded to the nearest multiple of ten. All proportions were calculated using these rounded counts.

Era	Total		No indication of infection		Interval between indication of infection and surgery							
					≤14 days		15-28 days		29-42 days		≥43 days	
	n / N	%	n / N	%	n / N	%	n / N	%	n / N	%	n / N	%
Pre-pandemic	2,470 / 1,918,850	0·1	-	-	-	-	-	-	-	-	-	-
Pandemic-no-vaccines	1,710 / 497,110	0·3	1,620 / 491,220	0·3	40 / 970	4·1	20 / 860	2·3	10 / 780	1·3	30 / 3,280	0·9
Pandemic-with-vaccines	2,480 / 1,242,180	0·2	2,200 / 1,121,490	0·2	40 / 3,660	1·1	30 / 5,480	0·5	20 / 6,250	0·3	190 / 105,300	0·2
COVIDSurg data collection period (OpenSAFELY)	150 / 67,580	0·2	104 / 66·980	0·2	Redacted	-	0 / 80	0·0	0 / 50	0·0	Redacted	-
COVIDSurg data collection period (COVIDSurg Collaborative)	2,151 / 140,231	1·5	1,973 / 137,104	1·4	104 / 1,138	9·1	32 / 461	6·9	18 / 326	5·5	24 / 1,202	2·0

## Data Availability

This study used unconsented data, queried and analysed *in situ* via a secure platform, under a study-specific approval. No individual-level data were downloaded by the research team and thus no data can be shared. However, all analytic code is publicly available.
