# Supplementary material for: Postoperative mortality and complications in patients with and without preoperative SARS-CoV2 infection: A service evaluation of 24 million linked records using OpenSAFELY
Source: Anaesthesia. Author manuscript; Available in PMC 2024 Jul 9. (PMC7616145; doi:10.1111/anae.16001)
Supplement: Supplementary Material [file EMS197251-supplement-Supplementary_Material.pdf]

Supplementary material

Supplementary material

Supplementary material 1 – Further details on methods

Study population

Our population of interest was patients who underwent surgery. The exact list of SNOMED-CT codes used to identify patients https://www.opencodelists.org/codelist/user/ciaranmci/surgery-covidsurg-replication-excluding-exclusions/5c09dd62/. To define 'surgery', we combined all codes in the SNOMED-CT tree pertaining to 'surgery' or 'surgical procedure' but excluded all terms in the COVIDSurg collaborative's list of excluded procedures. Using NHS Digital's mapping tables (Technology Reference Update Distribution, TRUD (1)), the resultant SNOMED-CT code list was converted to OPCS 4.9 and compared to the 'Intermediate' definition of surgery as described by Abbott et al. (2) to identify any missed procedure codes. The resultant combined list was then backwards-mapped to SNOMED-CT to conduct the patient records queries. All codelists that we used are indicated in the 'Methodology' section of the metadata for the surgery codelist, linked above. All codelists are publicly available at https://opencodelists.org for inspection and reuse by the wider research community.

Variable definitions and codelists

Table S1 shows the codelists used to inform the study's variables. Each weblink leads to an Opencodelist that explains the content and derivation of the codelist.

A note on demographic stratifications

With respect to the COVIDSurg study that we adapted (3), where COVIDSurg stratified on Revised Cardiac Risk Index, we stratified based on the presence of chronic cardiac disease and on the presence of diabetes; where COVIDSurg defined the presence of respiratory conditions as either asthma or chronic obstructive pulmonary disease, we define the presence of respiratory conditions as asthma or other chronic respiratory conditions; where COVIDSurg defined urgency of surgery as surgery on either emergency or elective admission, we retitled the variable as "Admission method" and defined it on the basis of NHS Hospital Episode Statistics (4). Surgeries under admissions that did not match emergency or elective NHS Hospital Episode Statistics codes were labelled "Unknown".

Table S1 Weblinks to codelists for each of the variables used in the study.			
**Concept**	**Use**	**Variable**	**Opencodelist**
Chronic cardiac disease	Demographic stratification	Chronic cardiac disease	https://www.opencodelists.org/codelist/opensafely/chronic-cardiac-disease-snomed/4ac64b59
Diabetes	Demographic stratification	Diabetes	https://www.opencodelists.org/codelist/opensafely/diabetes-snomed/7a3271cd
Chronic respiratory disease	Demographic stratification	Chronic respiratory disease	https://www.opencodelists.org/codelist/opensafely/chronic-respiratory-disease/69a34cc0
Cerebrovascular disease	Demographic stratification & Outcome	Cerebrovascular disease & 30-day post-operative cerebrovascular complication	https://www.opencodelists.org/codelist/user/alwynkotze/covidsurg-replication-tia-stroke/31204854
Pulmonary complications	Outcome	30-day post-operative pulmonary complications	https://www.opencodelists.org/codelist/user/alwynkotze/postop-pulmonary-complications-covidsurg/1ac39515
Cardiac complications	Outcome	30-day post-operative cardiac complications	https://www.opencodelists.org/codelist/user/alwynkotze/mace-snomed/4ce27b7f/

References

1. NHS Digital. SNOMED CT UK Clinical Edition, RF2 - new releases [Internet]. 2022 [cited 2022 May 18]. Available from: https://isd.digital.nhs.uk/trud/users/authenticated/filters/0/categories/26/items/101/releases
2. Abbott TEF, Fowler AJ, Dobbs TD, Harrison EM, Gillies MA, Pearse RM. Frequency of surgical treatment and related hospital procedures in the UK: A national ecological study using hospital episode statistics. Br J Anaesth [Internet]. 2017;119(2):249–57. Available from: http://dx.doi.org/10.1093/bja/aex137
3. COVIDSurg Collaborative, GlobalSurg Collaborative. Timing of surgery following SARS-CoV-2 infection: an international prospective cohort study. Anaesthesia. 2021;76(6):748–58.
4. NHS Digital. Hospital Episode Statistics Data Dictionary [Internet]. Available from: https://digital.nhs.uk/data-and-information/data-tools-and-services/data-services/hospital-episode-statistics/hospital-episode-statistics-data-dictionary

Supplementary material 2 - Demographic and outcome data for other eras studied

Table S2.1 shows that the average age of patients undergoing surgery was similar in any of the eras studied, and that ages were widely spread with a minor skew to lower ages. The spread and skew are visible in the histogram plots of ages for each era shown in Figure S2.1.

Table S2.1 Descriptive statistics of the age distribution of cohorts from each era in the OpenSAFELY database. $\bar{x}$ = sample arithmetic mean; s = sample standard deviation.

Era	Arithmetic mean $\bar{x} = \frac{\sum_i x_i}{n}$	Standard deviation $s = \sqrt{\frac{\sum_i (x_i - \bar{x})^2}{n-1}}$	Skewness $\frac{\sum_i (x_i - \bar{x})^3}{n \cdot s^3}$	Excess kurtosis $\frac{\sum_i (x_i - \bar{x})^4}{n \cdot s^4} - 3$
Pre-pandemic	53·0	22·7	-0·4	-0·7
Pandemic no vaccine	52·4	22·9	-0·3	-0·8
Pandemic with vaccine	55·1	22·4	-0·5	-0·7
COVIDSurg data collection period	53·4	22·6	-0·4	-0·7

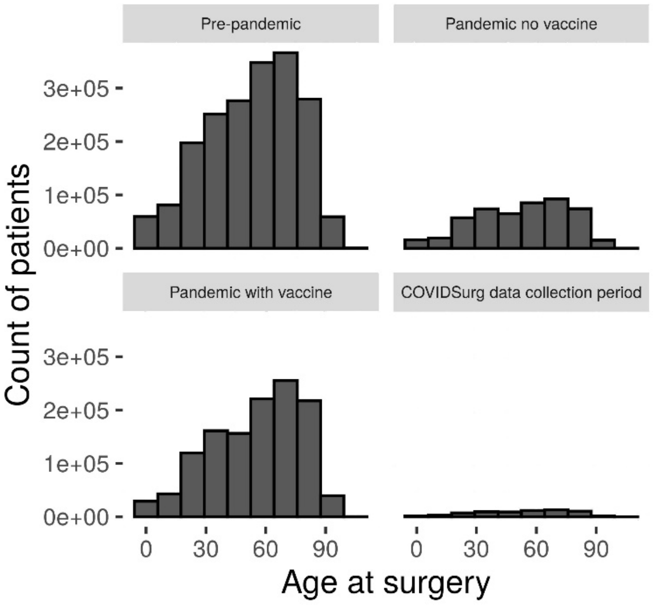

Figure S2.1 Histogram of ages for each of the four eras studied. The height of columns indicates the count of patients in each 10-year age band. The differences in height indicate the different count of patients that underwent surgery in each era, which was a function of the duration of each era: Pre-pandemic = 731 days; Pandemic no vaccine = 300 days; Pandemic with vaccine = 429; COVIDSurg data collection period = 27 days.

Tables S2.2 to S2.7 show the demographic characteristics and outcomes for patients who underwent surgery in each of the eras studied.

Table S2.2 Demographic characteristics for patients who underwent surgery stratified by duration from indication of SARS-CoV-2 infection to surgery date. Period of interest is from 17th March 2018-17th March 2020 (i.e. the *pre-pandemic* era). Values are counts (n) and percentages (%). In accordance with guidance from OpenSAFELY, all counts ≤7 were redacted before all remaining counts were rounded to the nearest multiple of ten. All proportions were calculated using these rounded counts.

		**No indication of infection**	**Interval between indication of infection and surgery**							
			**≤14 days**		**15-28 days**		**29-42 days**		**≥43 days**	
		(n = 1,918,850)	(n = -)		(n = -)		(n = -)		(n = -)	
		n (%)	n (%)		n (%)		n (%)		n (%)	
Sex										
	Female	1,050,950 (54·8%)	-	-	-	-	-	-	-	-
	Male	867,900 (45·2%)	-	-	-	-	-	-	-	-
Chronic cardiac disease										
	Yes	220,870 (11·5%)	-	-	-	-	-	-	-	-
	No	1,642,830 (88·5%)	-	-	-	-	-	-	-	-
Diabetes										
	Yes	276,030 (14·4%)	-	-	-	-	-	-	-	-
	No	1,642,830 (85·6%)	-	-	-	-	-	-	-	-
Chronic respiratory disease										
	Yes	119,410 (6·2%)	-	-	-	-	-	-	-	-
	No	1,799,450 (93·8%)	-	-	-	-	-	-	-	-
Cerebrovascular disease										
	Yes	73,420 (3·8%)	-	-	-	-	-	-	-	-
	No	1,845,440 (96·2%)	-	-	-	-	-	-	-	-
Admission method										
	Elective	1,112,730 (58·0%)	-	-	-	-	-	-	-	-
	Emergency	25,750 (1·3%)	-	-	-	-	-	-	-	-
	Unknown	780,370 (40·7%)	-	-	-	-	-	-	-	-

Table S2.3 Outcomes for patients who underwent surgery stratified by duration from indication of SARS-CoV-2 infection to surgery date. Period of interest is from 17th March 2018-17th March 2020 (i.e. the *pre-pandemic* era). Values are counts (n) and percentages (%). In accordance with guidance from OpenSAFELY, all counts ≤7 were redacted before all remaining counts were rounded to the nearest multiple of ten. All proportions were calculated using these rounded counts.

	**No indication of infection**	**Interval between indication of infection and surgery**			
		**≤14 days**	**15-28 days**	**29-42 days**	**≥43 days**
	(n = 1,918,850)	(n = -)	(n = -)	(n = -)	(n = -)
	n (%)	n (%)	n (%)	n (%)	n (%)
30-day post-operative mortality					
Alive within 30 days	1,916,390 (99·9%)	- -	- -	- -	- -
Dead within 30 days	2,470 (0·1%)	- -	- -	- -	- -
6-month post-operative mortality					
Alive within 6 months	1,899,170 (99·0%)	- -	- -	- -	- -
Dead within 6 months	19,680 (1·0%)	- -	- -	- -	- -
30-day post-operative pulmonary complications					
No complications	1,915,580 (99·8%)	- -	- -	- -	- -
Complications	3,280 (0·2%)	- -	- -	- -	- -
30-day post-operative cardiac complications					
No complications	1,902,500 (99·1%)	- -	- -	- -	- -
Complications	16,360 (0·9%)	- -	- -	- -	- -
30-day post-operative cerebrovascular complication					
No complications	1,917,120 (99·9%)	- -	- -	- -	- -
Complications	1,730 (0·1%)	- -	- -	- -	- -

Table S2.4 Demographic characteristics for patients who underwent surgery stratified by duration from indication of SARS-CoV-2 infection to surgery date. Period of interest is from 18th March 2020-12th Jan 2021 (i.e. the *pandemic-no-vaccines* era). Values are counts (n) and percentages (%). In accordance with guidance from OpenSAFELY, all counts ≤7 were redacted before all remaining counts were rounded to the nearest multiple of ten. All proportions were calculated using these rounded counts.

	**No indication of infection**	**Interval between indication of infection and surgery**			
		**≤14 days**	**15-28 days**	**29-42 days**	**≥43 days**
	(n = 491,220)	(n = 970)	(n = 860)	(n = 780)	(n = 3,280)
	n (%)	n (%)	n (%)	n (%)	n (%)
Sex					
Female	282,960 (57·6%)	610 (62·9%)	480 (55·8%)	430 (55·1%)	1,930 (58·8%)
Male	208,260 (42·4%)	360 (37·1%)	380 (44·2%)	340 (43·6%)	1,350 (41·2%)
Chronic cardiac disease					
Yes	58,070 (11·8%)	140 (14·4%)	140 (16·3%)	110 (14·1%)	510 (15·5%)
No	433,150 (88·2%)	830 (85·6%)	720 (83·7%)	670 (85·9%)	2,770 (84·5%)
Diabetes					
Yes	77,440 (15·8%)	220 (22·7%)	180 (20·9%)	140 (17·9%)	710 (21·6%)
No	413,780 (84·2%)	7750 (77·3%)	680 (79·1%)	630 (80·8%)	2,570 (78·4%)
Chronic respiratory disease					
Yes	30,720 (6·3%)	90 (9·3%)	70 (8·1%)	60 (7·7%)	270 (8·2%)
No	460,500 (93·7%)	880 (90·7%)	790 (91·9%)	720 (92·3%)	3,020 (92·1%)
Cerebrovascular disease					
Yes	19,480 (4·0%)	60 (6·2%)	50 (5·8%)	40 (5·1%)	220 (6·7%)
No	471,740 (96·0%)	910 (93·8%)	810 (94·2%)	740 (94·9%)	3,060 (93·3%)
Admission method					
Elective	263,040 (53·5%)	310 (32·0%)	360 (41·9%)	360 (46·2%)	1,590 (48·5%)
Emergency	9,400 (1·9%)	30 (3·1%)	30 (3·5%)	30 (3·8%)	80 (25·4%)
Unknown	218,770 (44·5%)	620 (63·9%)	480 (55·8%)	390 (50·0%)	1,610 (49·1%)

Table S2.5 Outcomes for patients who underwent surgery stratified by duration from indication of SARS-CoV-2 infection to surgery date. Period of interest is from 18th March 2020-12th Jan 2021 (i.e. the *pandemic-no-vaccines* era). Values are counts (n) and percentages (%). In accordance with guidance from OpenSAFELY, all counts ≤7 were redacted before all remaining counts were rounded to the nearest multiple of ten. All proportions were calculated using these rounded counts.

	**No indication of infection**	**Interval between indication of infection and surgery**			
		**≤14 days**	**15-28 days**	**29-42 days**	**≥43 days**
	(n = 491,220)	(n = 970)	(n = 860)	(n = 780)	(n = 3,280)
	n (%)	n (%)	n (%)	n (%)	n (%)
30-day post-operative mortality					
Alive within 30 days	489,600 (99·7%)	930 (95·9%)	840 (97·7%)	770 (98·7%)	3,260 (99·4%)
Dead within 30 days	1,620 (0·3%)	40 (4·1%)	20 (2·3%)	10 (1·3%)	30 (0·9%)
6-month post-operative mortality					
Alive within 6 months	481,780 (98·1%)	880 (90·7%)	810 (94·2%)	750 (96·2%)	3,140 (95·7%)
Dead within 6 months	9,440 (1·9%)	80 (8·2%)	50 (5·8%)	20 (2·6%)	140 (4·3%)
30-day post-operative pulmonary complications					
No complications	490,140 (99·8%)	940 (69·9%)	840 (97·7%)	760 (97·4%)	3,250 (99·1%)
Complications	1,080 (0·2%)	20 (2·1%)	20 (2·3%)	10 (1·3%)	40 (1·3%)
30-day post-operative cardiac complications					
No complications	486,100 (99·0%)	940 (96·9%)	830 (96·5%)	760 (97·4%)	3,230 (98·5%)
Complications	5,120 (1·0%)	30 (3·1%)	30 (3·5%)	20 (2·6%)	60 (1·8%)
30-day post-operative cerebrovascular complication					
No complications	490,650 (99·9%)	960 (99·0%)	860 (100·0%)	770 (98·7%)	3,270 (99·7%)
Complications	570 (0·1%)	Redacted	Redacted	Redacted	10 (0·3%)

Table S2.6 Demographic characteristics for patients who underwent surgery stratified by duration from indication of SARS-CoV-2 infection to surgery date. Period of interest is from 5th October 2020-1st November 2020 (i.e. the *COVIDSurg data collection period* era). Values are counts (n) and percentages (%). In accordance with guidance from OpenSAFELY, all counts ≤7 were redacted before all remaining counts were rounded to the nearest multiple of ten. All proportions were calculated using these rounded counts.

	No indication of infection	Interval between indication of infection and surgery			
		**≤14 days**	**15-28 days**	**29-42 days**	**≥43 days**
	(n = 66,980)	(n = 110)	(n = 80)	(n = 50)	(n = 360)
	n (%)	n (%)	n (%)	n (%)	n (%)
Sex					
Female	38,020 (56·8%)	70 (63·6%)	40 (50·0%)	30 (60·0%)	240 (66·7%)
Male	28,960 (43·2%)	50 (45·5%)	40 (50·0%)	20 (40·0%)	130 (36·1%)
Chronic cardiac disease					
Yes	8,040 (12·0%)	10 (9·1%)	10 (12·5%)	Redacted	60 (16·7%)
No	58,940 (88·0%)	100 (90·9%)	70 (87·5%)	40 (80·0%)	300 (83·3%)
Diabetes					
Yes	10,700 (16·0%)	20 (18·2%)	10 (12·5%)	Redacted	80 (22·2%)
No	56,280 (84·4%)	100 (90·9%)	70 (87·5%)	40 (80·0%)	208 (77·8%)
Chronic respiratory disease					
Yes	4,230 (6·3%)	Redacted	Redacted	Redacted	30 (8·3%)
No	62,750 (93·7%)	110 (100·0%)	80 (100·0%)	50 (100·0%)	330 (91·7%)
Cerebrovascular disease					
Yes	2,670 (4·0%)	Redacted	Redacted	Redacted	30 (8·3%)
No	64,310 (96·0%)	110 (100·0%)	80 (100·0%)	50 (100·0%)	340 (94·4%)
Admission method					
Elective	38,160 (5·7%)	50 (45·5%)	40 (50·0%)	30 (60·0%)	160 (44·4%)
Emergency	1,120 (1·7%)	Redacted	Redacted	0 (0·0%)	10 (2·8%)
Unknown	27,700 (41·4%)	60 (54·5%)	40 (50·0%)	20 (40·0%)	200 (55·6%)

Table S2.7 Outcomes for patients who underwent surgery stratified by duration from indication of SARS-CoV-2 infection to surgery date. Period of interest is from 5th October 2020-1st November 2020 (i.e. the *COVIDSurg data collection period* era). Values are counts (n) and percentages (%). In accordance with guidance from OpenSAFELY, all counts ≤7 were redacted before all remaining counts were rounded to the nearest multiple of ten. All proportions were calculated using these rounded counts.

	**No indication of infection**	**Interval between indication of infection and surgery**			
		**≤14 days**	**15-28 days**	**29-42 days**	**≥43 days**
	(n = 66,980)	(n = 110)	(n = 80)	(n = 50)	(n = 360)
	n (%)	n (%)	n (%)	n (%)	n (%)
30-day post-operative mortality					
Alive within 30 days	66,830 (99·8%)	110 (100·0%)	80 (100·0%)	50 (100·0%)	360 (100·0%)
Dead within 30 days	140 0·2%)	Redacted	0 (0·0%)	0 (0·0%)	Redacted
6-month post-operative mortality					
Alive within 6 months	65,870 (98·3%)	110 (100·0%)	80 (100·0%)	50 (100·0%)	340 (94·4%)
Dead within 6 months	1,110 (1·7%)	Redacted	Redacted	0 (0·0%)	20 (5·6%)
30-day post-operative pulmonary complications					
No complications	66,860 (99·8%)	110 (100·0%)	80 (100·0%)	50 (100·0%)	360 (100·0%)
Complications	120 (0·2%)	Redacted	Redacted	0 (0·0%)	Redacted
30-day post-operative cardiac complications					
No complications	66,370 (99·1%)	110 (100·0%)	80 (100·0%)	50 (100·0%)	360 (100·0%)
Complications	610 (0·9%)	Redacted	Redacted	Redacted	Redacted
30-day post-operative cerebrovascular complication					
No complications	66,910 (99·9%)	110 (100·0%)	80 (100·0%)	50 (100·0%)	360 (100·0%)
Complications	70 (0·1%)	0 (0·0%)	0 (0·0%)	0 (0·0%)	Redacted
